# Impact of visual impairment severity on working memory and executive function in children and adolescents

**DOI:** 10.3389/fpsyg.2026.1827656

**Published:** 2026-05-20

**Authors:** Kemily Correia, Tiago S. Bara, Vanessa Furlin, Carolina Prando, Perola G. Iankilevich, Mara L. Cordeiro

**Affiliations:** 1 Faculdades Pequeno Principe, Curitiba, Brazil; 2 Instituto de Pesquisa Pelé Pequeno Príncipe, Curitiba, Brazil; 3 Hospital Pequeno Principe, Curitiba, Brazil; 4 Department of Psychiatry and Biobehavioral Sciences, University of California Los Angeles, Los Angeles, CA, United States

**Keywords:** adaptive behavior, executive function, neurodevelopment, verbal learning, visual impairment, working memory

## Abstract

**Objective:**

Visual impairment (VI) affects millions of children worldwide and may influence neurodevelopmental processes beyond sensory functioning. Although cross-modal plasticity has been proposed as a compensatory mechanism, the extent to which VI severity affects verbal learning and executive functioning during development remains unclear. This study examined verbal and executive function profiles in children and adolescents with different degrees of VI.

**Methods:**

Eighty-six children and adolescents with VI, aged 6–16 years, were assessed using standardized measures. Verbal comprehension and working memory were evaluated with the Wechsler Intelligence Scale for Children - Fourth Edition (WISC-IV). Executive functioning was assessed through caregiver reports using the Behavior Rating Inventory of Executive Function (BRIEF), and auditory-verbal memory was explored using the Rey Auditory Verbal Learning Test (RAVLT). Participants were categorized according to VI severity: mild-to-moderate versus severe-to-profound. Group differences were analyzed using Mann–Whitney U tests, with statistical significance set at *p* < 0.05.

**Results:**

Participants with severe-to-profound VI showed significantly lower Verbal Comprehension Index scores in both childhood and adolescence compared with those with mild-to-moderate VI. In adolescence, greater VI severity was also associated with significantly poorer adaptive functioning across communication, daily living skills, socialization, and the overall Adaptive Behavior Composite. Group differences in caregiver-reported executive functioning and Working Memory Index scores did not reach statistical significance. A group difference was observed in a specific auditory-verbal learning measure during adolescence, with higher scores in the severe-to-profound VI group. However, this finding should be interpreted cautiously as task-specific.

**Conclusion:**

VI severity is associated with differences in cognitive and adaptive functioning in pediatric populations, with a particularly consistent association with verbal comprehension and age-dependent effects on adaptive behavior. These findings highlight the importance of considering severity levels in neurodevelopmental assessment and in the design of targeted educational and rehabilitative strategies.

## 1 Introduction

Visual impairment (VI) refers to a reduction in visual acuity or visual field that limits an individual’s ability to perceive visual stimuli, as defined by the International Classification of Diseases (ICD-10). In Brazil, approximately seven million people live with some degree of VI (IBGE - Instituto Brasileiro De Geografia E Estatística, 2020), and global projections indicate an increasing prevalence due to demographic and environmental factors. Beyond sensory loss, VI influences neurodevelopmental trajectories, affecting communication, learning, and social interaction from early childhood (Tadić et al., 2010; Bathelt et al., 2019).

Language acquisition and cognitive development in children with VI rely predominantly on auditory, tactile, and proprioceptive input. According to the Visual Mediation Hypothesis (Bruns and Röder, 2023), vision functions as a primary integrator by providing a simultaneous spatial representation that organizes multisensory information. In the absence of this visual anchor, the developing brain must rely more heavily on serial, temporally sequenced processing (e.g., auditory input). This shift may increase demands on working memory and executive control systems (Vitali and Gori, 2026). Together, this framework suggests that visual input plays a central role in reducing cognitive load during information integration, and that its absence may disproportionately tax higher-order cognitive processes.

These alternative sensory pathways contribute to distinct neurodevelopmental trajectories compared to sighted peers. Early visual deprivation may delay symbolic play, vocabulary expansion, and social referencing, as visual cues such as facial expressions and gaze are central to early communication (Sonksen and Dale, 2002; Argyropoulos et al., 2020). As a result, difficulties in interpreting social and emotional information may overlap with features observed in neurodevelopmental conditions, potentially complicating differential diagnosis.

Prior findings in this field can be broadly organized along two axes: developmental stage and cognitive domain. Studies in younger children tend to report delays in language-based symbolic processing, whereas adolescents often demonstrate relative preservation of verbal abilities but persistent difficulties in higher-order executive organization (Pring, 2008). Across domains, individuals with VI may exhibit relative strengths in auditory memory and verbal reasoning, possibly reflecting cross-modal neuroplasticity and cortical reorganization (Bathelt et al., 2019). However, these strengths frequently coexist with vulnerabilities in executive functions, particularly in working memory, inhibition, and planning (Tassé et al., 2016). This structured variability highlights the importance of identifying factors that contribute to heterogeneity in cognitive outcomes.

Within this framework, the severity of visual impairment represents a critical yet underexplored factor. Children with residual vision may use visual input as a scaffold for spatial mental representations, whereas those with severe or absent vision rely more heavily on cross-modal compensatory mechanisms. This increased reliance may elevate cognitive load and place greater demands on executive resources (Bathelt et al., 2019). Thus, VI severity may modulate the balance between compensatory neuroplasticity and cognitive demand.

Although cognitive functioning in children with VI has been widely investigated, the specific influence of VI severity on verbal and executive domains remains insufficiently characterized (Heyl and Hintermair, 2015). Many studies treat VI as a homogeneous condition, potentially obscuring meaningful variability associated with the degree of visual loss (Morelli et al., 2022; Jammal et al., 2023; Celik Turan and Aki, 2025). Clarifying whether cognitive profiles differ across severity levels is essential for understanding the limits of neuroplastic compensation across development. It also informs the design of targeted intervention strategies.

Therefore, the present study aimed to compare verbal comprehension, working memory, and executive functioning in children and adolescents with mild-to-moderate versus severe-to-profound visual impairment. We hypothesized that greater VI severity would be associated with increased cognitive load, reflected in lower working memory performance and greater executive difficulties. We further expected more pronounced metacognitive regulation deficits among participants with severe-to-profound impairment. Clarifying these differences may contribute to more precise neurodevelopmental assessment and inform the development of targeted educational and rehabilitative interventions.

## 2 Methods

### 2.1 Study design

This cross-sectional observational study was conducted to investigate the association between visual impairment (VI) severity and cognitive-executive functioning in children and adolescents. Data were collected individually at the Pelé Pequeno Príncipe Research Institute using standardized psychological assessments administered under controlled conditions.

### 2.2 Participants

The sample comprised 86 children and adolescents aged 6 to 16 years with a clinical diagnosis of visual impairment or low vision. Participants were recruited from the ophthalmology outpatient clinic of Pequeno Príncipe Hospital, a tertiary pediatric referral center, using a convenience sampling approach that aimed to include all eligible patients during the study period.

Participants were eligible if they: (1) had a diagnosis of visual impairment established by a specialized ophthalmologist; (2) were between 6 and 16 years of age; and (3) were able to complete the assessment procedures. Exclusion criteria included: (1) severe neurological conditions that prevented participation in cognitive testing; (2) uncorrected hearing impairment; and (3) insufficient clinical or ophthalmological data.

The diagnosis of visual impairment was established by ophthalmologists based on clinical evaluation, including best-corrected visual acuity (BCVA), following criteria defined by Brazilian legislation (Decree No. 5.296/2004), which classifies low vision as visual acuity between 0.3 (20/60) and 0.05 (20/400), and blindness as visual acuity equal to or less than 0.05 (20/400) in the better eye, with best optical correction.

Given the clinical nature of the sample, participants presented heterogeneous etiologies of visual impairment, including isolated ocular conditions, infectious causes, and neurodevelopmental or genetic syndromes. Comorbid conditions were not used as exclusion criteria, reflecting the complexity of cases typically observed in tertiary referral settings.

Socioeconomic status (SES) was classified according to the Brazilian Economic Classification Criteria (ABEP), allowing characterization of the sample’s socioeconomic distribution.

To address developmental heterogeneity, participants were stratified into two age groups: childhood (6–9 years) and adolescence (10–16 years), based on established non-linear trajectories of neurocognitive maturation.

#### 2.2.1 Visual impairment assessment and classification

Visual impairment (VI) severity was determined based on best-corrected visual acuity (BCVA) in the better-seeing eye, obtained from participants’ ophthalmological records. All participants had a prior clinical diagnosis established by specialized ophthalmologists, following standard age-appropriate procedures, including visual acuity assessment using Snellen or equivalent charts.

Visual acuity values (e.g., 20/60, 20/200, 20/400) are expressed according to the Snellen scale and represent the distance at which a participant can correctly identify optotypes compared to an individual with normal vision.

Classification of VI severity followed internationally recognized criteria, including World Health Organization (2006) recommendations and ICD-aligned thresholds, based on BCVA in the better eye:

- Normal vision: > 0.8 (approximately 20/25 or better)
- Mild visual impairment: < 0.8 and ≥ 0.3 (approximately 20/60)
- Moderate visual impairment: < 0.3 and ≥ 0.125 (approximately 20/60 to 20/200)
- Severe visual impairment: < 0.125 and ≥ 0.02 (worse than 20/200 to 20/400)
- Blindness: < 0.02 or presence of only light perception (LP), corresponding to vision worse than 20/400
- Amaurosis: absence of light perception (no light perception; NLP)

For analytical purposes, participants were categorized into two groups:

- Mild-to-moderate VI (visual acuity between <20/60 and 20/200)
- Severe-to-profound VI (visual acuity ≤20/400, including blindness and absence of light perception)

This classification enabled comparison of cognitive and functional outcomes across clinically meaningful levels of VI severity.

Given the clinical nature of the sample, visual impairment etiologies were highly heterogeneous, reflecting the complexity of a tertiary pediatric referral population. Etiologies included isolated congenital ocular conditions (e.g., congenital glaucoma, cataracts), infectious sequelae (e.g., congenital toxoplasmosis, syphilis), optic pathway and neurological anomalies (e.g., optic atrophy, septo-optic dysplasia), severe refractive and oculomotor disorders (e.g., high myopia, strabismus, nystagmus), and genetic or neurodevelopmental syndromes (e.g., Joubert syndrome, Cockayne syndrome, Noonan syndrome, Bardet–Biedl syndrome, and mucopolysaccharidoses).

Socioeconomic status (SES) was classified according to the Brazilian Economic Classification Criteria (ABEP). The sample predominantly comprised families from middle-to-low socioeconomic backgrounds (primarily classes C and D/E), with a smaller proportion from higher socioeconomic strata (classes A and B). Access to educational support varied across participants, ranging from regular schooling without accommodations to specialized educational services.

All procedures were explained to caregivers and participants prior to data collection. Written informed consent was obtained from legal guardians, and assent was obtained from minors. The study was approved by the Brazilian National Research Ethics Committee on Human Research (CONEP; approval number: 4.195.697).

To account for developmental heterogeneity, participants were stratified into two age groups: childhood (6–9 years) and adolescence (10–16 years), based on established non-linear trajectories of neurocognitive maturation (Arain et al., 2013; Cowan, 2022; Tervo-Clemmens et al., 2023; Yang et al., 2023).

### 2.3 Measures

#### 2.3.1 Wechsler intelligence scale for children – fourth edition (WISC-IV)

The Wechsler Intelligence Scale for Children – Fourth Edition (WISC-IV) was used to assess cognitive functioning in children aged 6 to 16 years. To minimize confounding effects related to visual processing demands, only verbally mediated subtests were administered (Similarities, Vocabulary, Comprehension, Digit Span, and Letter-Number Sequencing). Composite scores were derived for the Verbal Comprehension Index and Working Memory Index, both of which are standardized according to age-based normative data (Wechsler, 2013).

#### 2.3.2 Behavior rating inventory of executive function (BRIEF)

Executive functioning was assessed using the Behavior Rating Inventory of Executive Function (BRIEF), a standardized caregiver-report instrument that evaluates executive behaviors in everyday contexts for individuals aged 5 to 18 years. The BRIEF provides two primary indices: the Behavioral Regulation Index (inhibitory control, flexibility, and emotional regulation) and the Metacognition Index (planning, organization, monitoring, and working memory). Higher scores indicate greater executive dysfunction (Gioia, 2000; Miranda and Bueno, 2011).

#### 2.3.3 Rey auditory verbal learning test (RAVLT)

Auditory-verbal memory was assessed using the Rey Auditory Verbal Learning Test (RAVLT), which evaluates immediate recall, learning across trials, and delayed recall through repeated presentation of a word list. This measure was included to explore potential cross-modal compensatory mechanisms in verbal learning among participants with visual impairment (Diniz, 2000).

#### 2.3.4 Vineland adaptive behavior scales – third edition (Vineland-3)

Adaptive functioning was assessed using the Vineland Adaptive Behavior Scales – Third Edition (Vineland-3), based on caregiver report. The instrument evaluates performance across key domains, including Communication, Daily Living Skills, and Socialization, and provides an overall Adaptive Behavior Composite score reflecting age-normed functioning (Sparrow et al., 2019).

### 2.4 Procedure

Following informed consent from legal guardians and assent from participants, all assessments were conducted individually at the Pelé Pequeno Príncipe Research Institute.

Cognitive and memory assessments (WISC-IV and RAVLT) were administered by trained psychologists, with verbal instructions adapted as needed to accommodate participants’ visual limitations. Sessions lasted approximately 60 min and were conducted in a quiet room with minimal visual stimuli to facilitate mobility and reduce environmental distraction.

After rapport was established, three verbally mediated subtests contributing to the Verbal Comprehension Index and two subtests contributing to the Working Memory Index of the WISC-IV were administered, followed by the RAVLT.

Caregiver-report instruments (BRIEF and Vineland-3) were completed in parallel in a separate room, independently whenever possible and under professional supervision, when necessary, with an approximate duration of 45 min.

### 2.5 Data analysis

Data distribution was assessed using the Shapiro–Wilk test. As several variables did not meet normality assumptions, non-parametric analyses were performed.

Descriptive statistics (means, standard deviations, and frequencies) were calculated and reported separately for each age group. Group differences between mild-to-moderate and severe-to-profound VI were examined using Mann–Whitney U tests within each age stratum. Effect sizes were calculated using the formula *r = Z/√N*.

To estimate the clinical risk associated with VI severity, Odds Ratios (OR) with 95% confidence intervals (CI) were calculated. For this analysis, continuous variables were categorized based on established clinical thresholds: scores below 70 were classified as impaired for WISC-IV indices, and T-scores ≥ 65 indicated clinically significant dysfunction for the BRIEF.

All statistical analyses were conducted using SPSS version 21.0 (IBM Corp., Armonk, NY, USA), with the alpha level set at *p* < 0.05.

## 3 Results

### 3.1 Sample characteristics

The total sample comprised 86 participants, stratified into children (6–9 years; *N* = 39) and adolescents (10–16 years; *N* = 47) to account for developmental heterogeneity. Demographic and clinical characteristics, presented separately by age group and visual impairment (VI) severity, are shown in Table 1.

**Table 1:** Demographic data stratified by age group and visual impairment severity.

Variable	Mild-to-moderate VI (*N* = 37)	Severe-to-profound VI (*N* = 49)	*p*
Childhood (6–9 years)	(*N* = 17)	(*N* = 22)	
Age (years), Mean (SD)	7.35 (0.86)	7.55 (1.26)	> 0.05
Sex (Boys), *n* (%)	13 (76.5%)	11 (50.0%)	—
Sex (Girls), *n* (%)	4 (23.5%)	11 (50.0%)	—
Adolescence (10–16 years)	(*N* = 20)	(*N* = 27)	
Age (years), Mean (SD)	13.62 (2.16)	12.96 (2.21)	> 0.05
Sex (Boys), *n* (%)	10 (50.0%)	12 (44.4%)	—
Sex (Girls), *n* (%)	10 (50.0%)	15 (55.6%)	—

### 3.2 Cognitive, executive and adaptive outcomes in childhood (6–9 years old)

The childhood cohort included 17 participants with mild-to-moderate VI and 22 with severe-to-profound VI. No significant age differences were observed between groups (mild-to-moderate: *M* = 7.35, *SD* = 0.86; severe-to-profound: *M* = 7.55, *SD* = 1.26; *p* > 0.05).

In terms of cognitive performance (WISC-IV), children with severe-to-profound VI demonstrated significantly lower Verbal Comprehension Index scores compared to those with mild-to-moderate VI (*U* = 38.5, *z* = −2.45, *p* = 0.014). No statistically significant group differences were observed for the Working Memory Index (*p* = 0.327).

Parent-reported executive functioning (BRIEF) and adaptive functioning (Vineland-3) did not differ significantly between groups across any domains in this age cohort (all *p* > 0.05; see Table 2).

**Table 2:** Cognitive, executive and adaptive performance data stratified by age group.

Variable	Mild-to-moderate VI (*N* = 37)	Severe-to-profound VI (*N* = 49)	*p*
Cognitive (WISC- IV)			
Childhood (6–9 years)	(*N* = 17)	(*N* = 22)	
Verbal comprehension index, Mean (SD)	89.82 (20.06)	65.69 (26.13)	0.014*
Working memory index, Mean (SD)	77.15 (17.15)	68.80 (29.59)	0.327
Adolescence (10–16 years)	(*N* = 20)	(*N* = 27)	
Verbal Comprehension Index, Mean (SD)	81.40 (18.01)	60.71 (18.80)	0.002*
Working Memory Index, Mean (SD)	76.73 (19.78)	64.89 (27.07)	0.233
Executive (BRIEF)			
Childhood (6–9 years)	(*N* = 17)	(*N* = 22)	
Behavioral regulation index, Mean (SD)	60.63 (15.66)	52.50 (12.88)	0.147
Metacognition index, Mean (SD)	62.06 (15.44)	57.88 (14.01)	0.439
Global executive composite, Mean (SD)	63.06 (16.97)	56.25 (12.91)	0.258
Adolescence (10–16 years)	(*N* = 20)	(*N* = 27)	
Behavioral regulation index, Mean (SD)	56.61 (15.52)	61.75 (12.30)	0.308
Metacognition index, Mean (SD)	60.06 (14.02)	57.94 (10.79)	0.654
Global executive composite, Mean (SD)	59.11 (14.71)	59.87 (10.83)	0.796
Adaptive (VINELAND)			
Childhood (6–9 years)	(*N* = 17)	(*N* = 22)	
Communication	74.00 (17.02)	63.25 (27.51)	>0.05
Daily living skills	75.42 (13.24)	65.94 (24.78)	>0.05
Socialization	78.00 (13.46)	70.67 (18.55)	>0.05
Adaptive behavior composite	74.83 (11.46)	68.19 (21.03)	>0.05
Adolescence (10–16 years)	(*N* = 20)	(*N* = 27)	
Communication	74.00 (21.43)	51.05 (29.43)	0.016*
Daily living skills	72.39 (25.34)	45.43 (25.59)	0.003*
Socialization	70.06 (17.43)	57.00 (19.84)	0.035*
Adaptive behavior composite	67.89 (19.25)	52.29 (22.19)	0.033*

^*^Statistical significance was defined as *p* < 0.05. WISC-IV indices and Vineland-3 domains are presented as Standard Scores (normative Mean = 100, SD = 15), whereas BRIEF indices are presented as T-scores (normative Mean = 50, SD = 10).

### 3.3 Cognitive, executive and adaptive outcomes in adolescence (10–16 years old)

The adolescent cohort comprised 20 participants with mild-to-moderate VI and 27 with severe-to-profound VI. No significant age differences were found between groups (*p* > 0.05).

Consistent with the childhood findings, adolescents with severe-to-profound VI demonstrated significantly lower Verbal Comprehension Index scores (*M* = 60.71, *SD* = 18.80) compared to those with mild-to-moderate VI (*M* = 81.40, *SD* = 18.01; *U* = 60.5, *z* = −3.15, *p* = 0.002). No significant differences were observed for the Working Memory Index (*p* = 0.233).

In contrast to the childhood cohort, adaptive functioning (Vineland-3) was significantly affected by VI severity during adolescence. Participants with severe-to-profound VI showed lower scores across all domains: Communication (*U* = 104.0, *z* = −2.40, *p* = 0.016), Daily Living Skills (*U* = 83.5, *z* = −2.99, *p* = 0.003), Socialization (*U* = 114.0, *z* = −2.11, *p* = 0.035), and Adaptive Behavior Composite (*U* = 113.5, *z* = −2.13, *p* = 0.033).

No significant group differences were observed in caregiver-reported executive functioning (BRIEF Global Executive Composite: *U* = 136.5, *p* = 0.796). (See Table 2).

Assessment of auditory-verbal memory (RAVLT) revealed a group difference in a specific verbal encoding measure during adolescence, with the severe-to-profound VI group showing higher scores than the mild-to-moderate group (*U* = 25.5, *z* = −2.16, *p* = 0.031). Given the absence of a typically developing control group and the domain-specific nature of this finding, this result should be interpreted cautiously as a task-specific difference rather than as evidence of a generalized compensatory advantage.

### 3.4 Risk analysis

Risk analyses were conducted to estimate the likelihood of cognitive, executive, and adaptive impairments associated with VI severity.

In the childhood cohort, participants with severe-to-profound VI showed significantly higher odds of impairment in the Verbal Comprehension Index (OR = 16.67, 95% CI [1.69, 164.79]) compared to those with mild-to-moderate VI. No significant increases in risk were observed for Working Memory (OR = 3.38, 95% CI [0.71, 16.17]), executive functioning (Global Executive Composite: OR = 0.21, 95% CI [0.04, 1.05]), or adaptive functioning domains (all CIs crossed 1).

In the adolescent cohort, participants with severe-to-profound VI also showed increased odds of impairment in the Verbal Comprehension Index (OR = 5.50, 95% CI [1.28, 23.69]). Although the Working Memory Index showed an elevated odds ratio (OR = 4.57), the confidence interval crossed the null value (95% CI [0.90, 23.14]).

Notably, significant increases in risk were observed for adaptive functioning domains during adolescence, including Daily Living Skills (OR = 6.40, 95% CI [1.57, 26.03]) and Socialization (OR = 3.93, 95% CI [1.03, 15.00]). No significant risk differences were observed for executive functioning or auditory-verbal memory measures.

## 4 Discussion

The present study examined whether the severity of visual impairment (VI) is associated with differences in cognitive, executive, and adaptive functioning in school-aged children and adolescents. Overall, the findings indicate that greater VI severity is consistently associated with lower Verbal Comprehension Index scores across developmental stages. During adolescence, greater severity was also associated with broader difficulties in adaptive functioning. In contrast, group differences in the Working Memory Index and caregiver-reported global executive functioning did not reach statistical significance.

Previous research has described enhanced verbal memory in some individuals with congenital blindness, often interpreted as reflecting cross-modal reorganization or compensatory strategy use (Arcos et al., 2022; Raz et al., 2007). At the same time, other studies have reported performance below age expectations in language-related or executive domains among children with VI, particularly when tasks involve higher demands on organization, processing efficiency, or socially mediated language use (Heyl and Hintermair, 2015; Greenaway et al., 2017).

The present findings do not support a generalized pattern of verbal compensation. Instead, we observed a difference in a specific RAVLT verbal encoding measure during adolescence. This finding may reflect the use of alternative learning strategies in some individuals with more severe VI. However, because verbal memory was not the primary outcome and no typically developing comparison group was included, this result should be interpreted cautiously. It likely reflects a task-specific effect rather than a generalized compensatory advantage.

The most robust cognitive finding was the lower Verbal Comprehension Index observed in the severe-to-profound VI group across both developmental stages. One plausible explanation is that reduced access to visually mediated experiences limits opportunities for incidental learning, concept formation, and the integration of language with contextual and spatial information over time (Sonksen and Dale, 2002; Bathelt et al., 2019; Bruns and Röder, 2023).

Within the framework of visual mediation, reduced or absent visual input may increase reliance on sequential processing. This shift may place greater demands on cognitive integration mechanisms (Bruns and Röder, 2023; Vitali and Gori, 2026).

Rather than implying a direct causal effect, the present findings suggest that greater VI severity is associated with increased vulnerability in domains that depend on cumulative developmental experience.

In contrast, although the severe-to-profound VI group showed numerically lower Working Memory Index scores, these differences were not statistically significant. This finding may indicate that verbally mediated working memory processes are relatively preserved under certain task conditions. Alternatively, variability within groups may reduce the ability to detect subtle effects. Previous studies have reported mixed findings in working memory performance among individuals with VI, suggesting that task characteristics and compensatory strategies may influence outcomes (Rindermann et al., 2020; Bathelt et al., 2019).

Regarding executive functioning, caregiver-reported measures (BRIEF) did not reveal significant group differences in either global or domain-specific indices. Although some variability was observed descriptively, the data do not support a clear association between VI severity and everyday executive functioning at the global level. Executive functioning in daily life may also be influenced by contextual and environmental factors, such as school accommodations, family support, and adaptive expectations (Greenaway et al., 2017; Heyl and Hintermair, 2015), which were not fully modeled in the present study.

Adaptive functioning showed the clearest developmental-stage effect. While no significant differences were observed in childhood, adolescents with severe-to-profound VI demonstrated consistently lower scores across communication, daily living skills, socialization, and the overall Adaptive Behavior Composite. This pattern may reflect the cumulative developmental impact of reduced access to incidental visual learning. The effect may become more pronounced during adolescence, a period characterized by increasing demands on autonomy, social integration, and independent functioning (Tadić et al., 2010; Papadopoulos et al., 2011; Pinquart and Pfeiffer, 2014). In this sense, VI severity appears to be associated not only with sensory limitation, but also with increasing challenges in meeting age-related adaptive expectations over time.

The risk analysis adds a clinically relevant dimension to these findings. Across both developmental stages, severe-to-profound VI was associated with increased odds of impairment in verbal comprehension. During adolescence, it was also associated with higher odds of deficits in daily living skills and socialization. Although these estimates should be interpreted cautiously given the sample size and the width of the confidence intervals, they reinforce the importance of considering VI severity as a meaningful factor in clinical assessment and intervention planning.

From a clinical and educational perspective, these findings support severity-sensitive and developmentally informed approaches to intervention. For children with severe-to-profound VI, early support may benefit from emphasizing explicit verbal mediation, structured environmental exploration, and reduced reliance on incidental visual learning (Morelli et al., 2022; Alves et al., 2009). During adolescence, intervention priorities may increasingly focus on promoting adaptive autonomy, social communication, and daily living skills. For individuals with residual vision, strategies may instead emphasize optimization of remaining visual function through environmental adaptations, assistive technologies, and multimodal instructional approaches (Perrault et al., 2023). Thus, VI severity may influence not only the level of support required, but also the type of intervention most likely to be effective.

This study has several limitations that should be considered. First, the cross-sectional design precludes causal inference and limits conclusions about developmental trajectories. Second, the use of convenience sampling in a specialized referral center may introduce referral bias and limit generalizability. Third, no *a priori* power analysis was conducted. In addition, subgroup sample sizes were modest following age stratification, which may have reduced sensitivity to detect smaller effects.

An additional limitation is the absence of a typically developing control group. This restricts the ability to determine whether the observed profiles reflect developmental delay, difference, or compensation relative to normative developmental trajectories. Furthermore, executive and adaptive functioning were assessed exclusively through caregiver-report measures (BRIEF and Vineland-3). Although these instruments provide ecologically valid information, they are subject to reporting bias and shared method variance. They also do not substitute for performance-based measures of executive functioning.

This limitation is particularly relevant given the scarcity of validated performance-based tools for children and adolescents with VI in the Brazilian context. Finally, the sample was heterogeneous with respect to VI etiology, socioeconomic background, educational support, and potential comorbid neurodevelopmental conditions. Although this reflects real-world clinical populations, the sample size did not allow for inclusion of these variables as covariates in the primary analyses. Future multicenter and longitudinal studies with larger and more stratified samples are needed to clarify developmental mechanisms and disentangle severity effects from contextual influences. In addition, multimethod assessment approaches may help identify the most effective intervention strategies across developmental stages.

## 5 Conclusion

This study examined the association between visual impairment (VI) severity and cognitive and adaptive functioning in school-aged children and adolescents. The findings indicate that greater VI severity is associated with lower verbal comprehension across development and, during adolescence, with increased vulnerability in adaptive functioning domains.

These results highlight the importance of incorporating VI severity into neurodevelopmental assessment and educational planning. Early identification of language-related and adaptive challenges may support more targeted and developmentally appropriate interventions. Interventions that emphasize structured multisensory experiences, explicit verbal mediation, and caregiver-supported learning environments may help mitigate the cumulative developmental impact associated with more severe visual impairment. Overall, VI severity emerges as a meaningful factor in explaining variability in cognitive and adaptive outcomes in pediatric populations.

## Data Availability

The original contributions presented in the study are included in the article/supplementary material, further inquiries can be directed to the corresponding author.
